# Sloth bear attacks: regional differences and safety messaging

**DOI:** 10.1038/s41598-022-07974-y

**Published:** 2022-03-10

**Authors:** Thomas R. Sharp, Tom S. Smith, Shanmugavelu Swaminathan, Attur S. Arun

**Affiliations:** 1 Wildlife SOS, 406 East 300 South, No. 302, Salt Lake City, UT 84111 USA; 2 Brigham Young University, 5050 Life Sciences Building, Provo, UT 84602 USA; 3 Bannerghatta Bear Rescue and Rehab Centre, Wildlife SOS, Bangalore, 560083 India

**Keywords:** Ecology, Evolution, Zoology, Ecology, Environmental social sciences

## Abstract

Sloth bears behave aggressively toward humans when threatened and are among the most dangerous wildlife in India. Safety messaging for those who live in sloth bear country must be accurate to be effective, and messaging may need to be modified to account for regional differences in human-bear relationships. The timing of sloth bear attacks on the Deccan Plateau of Karnataka, both by season and by time of day, deviated enough from those reported in other areas such that it warranted further investigation. We compared data from eight studies of human-sloth bear conflict from across the Indian subcontinent and explored possibilities as to why differences exist. Seasonally all studies reported that human-sloth bear conflict was highest when human activity in the forest was greatest, though the season of highest human activity varied significantly by region (*χ*^2^ = 5921, df = 5, *P* < 0.001). The time of day that the majority of attacks occurred also varied significantly by region (*χ*^2^ = 666, df = 5, *P* < 0.001), though human activity was relatively consistent. We speculated that the rate of day attacks on the Deccan Plateau was lower due to the reduced probability of encountering a sleeping bear as they are concealed and secure in shallow caves. Additionally, the rate of attacks was significantly higher at night on the Deccan Plateau because people often to work into nighttime. We concluded that slight differences, or different emphasis, to bear safety messaging may be necessary on a regional basis to keep the messaging accurate and effective.

## Introduction

The sloth bear (*Melursus ursinus*) is the most ubiquitous bear species in India and ranges throughout the subcontinent^1^. It is also considered one of the most dangerous wild animals in the region^2, 3^. Several studies have chronicled human-sloth bear conflict, and some have provided safety messaging advice intended to mitigate conflicts^4–6^. Additionally, sloth bear safety messaging has been dispensed through pamphlets, booklets, videos and workshops to reach those living and working in sloth bear country.

For bear safety messaging to be effective it must be accurate and generally it must be short and to the point. The basic rule ‘less is more’ applies to bear safety messaging as most people will not remember more than three or four key points^7^, and perhaps even less when under the duress associated with a sudden bear encounter. And while too much information overly complicates messaging, ignoring regional differences is ill-advised when differences exist. Alaska’s interior brown bears, for example, are significantly more aggressive than Alaska’s coastal brown bears^8^. The varying levels of aggressiveness are attributed to resource density and habituation with conspecifics and people. Consequently, Denali National Park advises people to maintain at least 400 m distance from brown bears whereas coastal Katmai recommends just 50 m as a safe distance^8^.

Bear safety messaging is based on bear behaviors that are both intrinsic and extrinsic to the species. For example, it is known that sloth bears, irrespective of location, have an innate defensive-aggressive response to surprise (sudden) encounters. This intrinsic response is likely due to having co-evolved with tigers, a formidable predator which opportunistically prey on sloth bears^9–12^. Alternately, coastal brown bears are more tolerant of other bears, and humans, than their interior counterparts likely due to being accustomed to a high density of bears in the region^8^. There have been calls to standardize bear safety messaging across the range of bear habitats^13^ but we suggest that a ‘one size fits all’ approach may seek consistency at the risk of safety. Recognizing that a bear’s response to human encounters often varies by region is key to providing effective bear safety messaging for people living in a specific area.

Our recent study^6^ of sloth bear attacks on the Deccan Plateau differed significantly from other studies of human-sloth bear conflict in terms of the timing of attacks, both by season and by time of day. While some variation is expected between areas, the differences between our study and others were substantial enough to warrant further investigation and for us to consider incorporating significant changes to the safety messaging for living with sloth bears on the Deccan Plateau. In this paper we present those differences and discuss their implications for effective bear safety messaging.

## Study area

**Figure 1:**
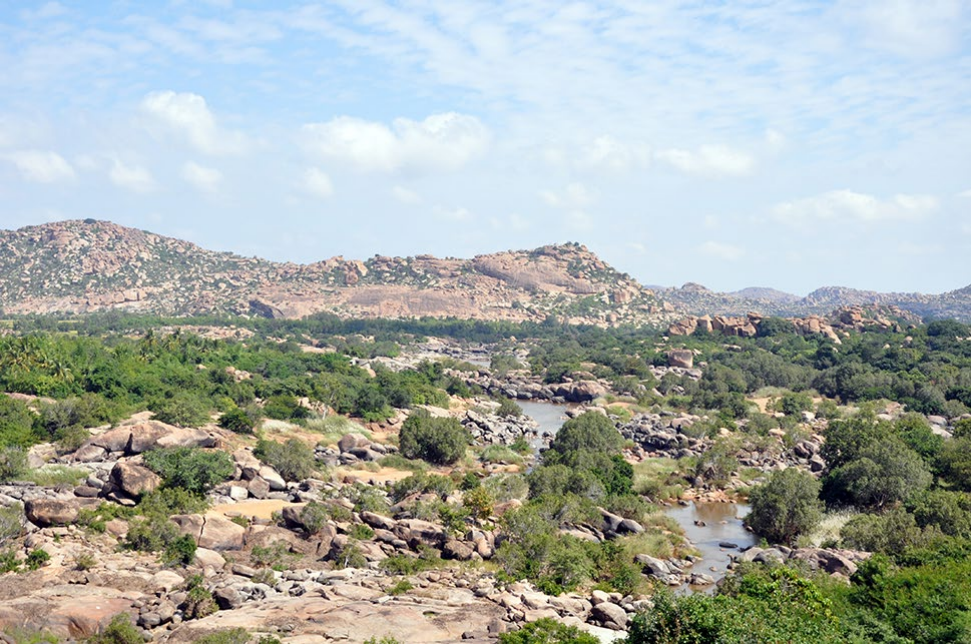
Sloth bear habitat on the Deccan Plateau.

We studied human-sloth bear conflict in the Indian state of Karnataka on the Deccan Plateau^6^. Sloth bear habitat on the Deccan Plateau is considered some of the highest quality for the species^14^. This area is largely comprised of rocky scrub forest with an abundance of naturally occurring caves (Fig. 1). The climate is semi-arid and characterized by hot summers (24°– 45 °C) during April–June and low rainfall (571–802 mm) from June to November^15^.

**Figure 2:**
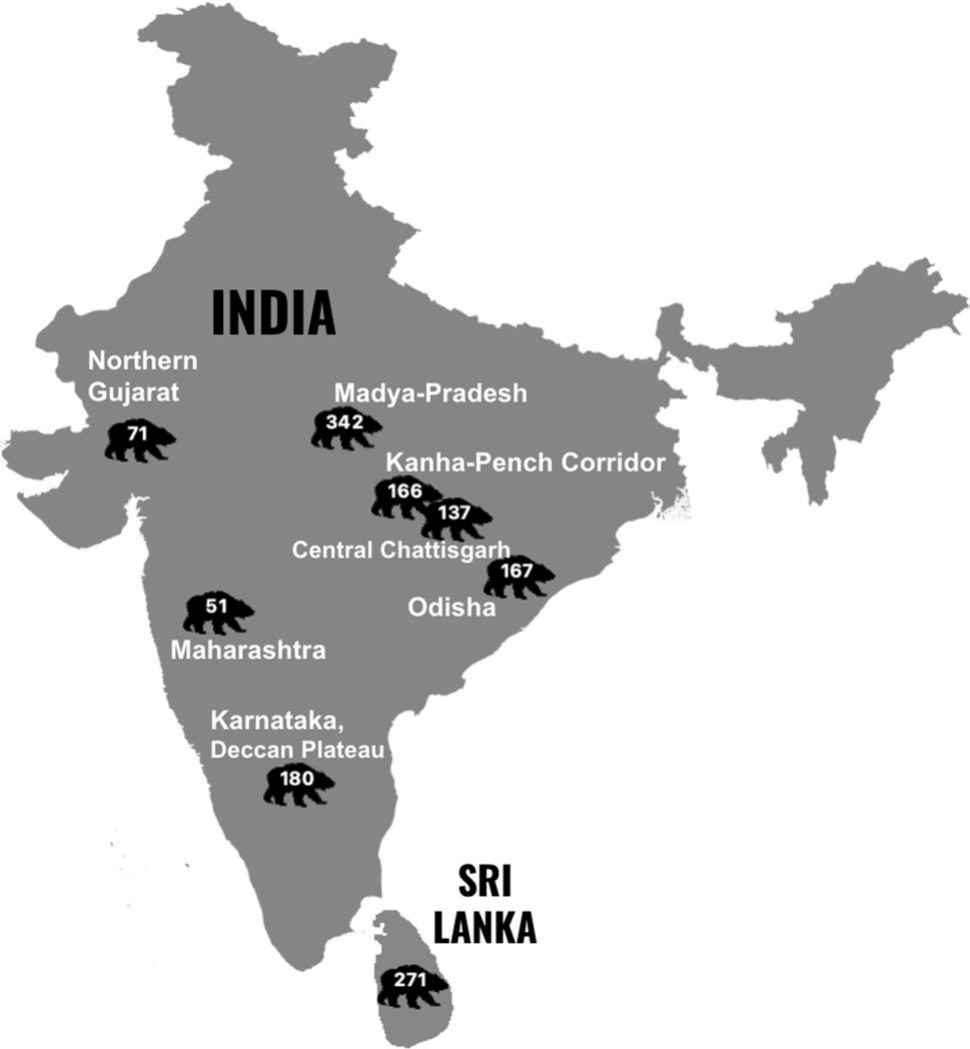
Sloth bear attack study locations and number of attacks (QGIS Geographic Information System. Version 3.14.0-Pi. QGIS Association. http://www.qgis.org).

For this work we compared the seasonal and diurnal timing of sloth bear conflicts on the Deccan Plateau^6^ to that reported in seven other studies, five of which were conducted in central India. Central India studies occurred in the states of Chhattisgarh, Odisha, Madya Pradesh, and Maharashtra. Additionally, one study was conducted in the state of Gujarat (west-central) and another in Sri Lanka (Fig. 2).

## Methods

We compared the temporality of bear attacks on the Deccan Plateau, both seasonally and within a 24-h period, to that reported in studies presented in Table 1.

**Table 1:** Sloth bear attack studies.

Location	Years	# of Attacks	Authors	Published
Madya Pradesh	1989–1994	735	Rajpurohit and Krausman	2000
Chhattisgarh	1998–2000	137	Bargali et al	2005
Sri Lanka	1938–2004	271	Ratnayeke et al	2014
Gujarat	2008–2009	71	Garcia et al	2016
Odisha	2002–2013	167	Debata et al	2016
Kanha–Pench corridor	2004–2016	166	Dhamorikar et al	2017
Maharashtra	2009–2017	51	Singh et al	2018
Deccan Plateau	1985–2916	180	Sharp et al	2020

Since the definition of seasons varied between studies, we standardized all seasonal data as follows: (1) summer, (2) monsoon and (3) winter. Sri Lanka is generally characterized as having four seasons, not three, two of which are characterized as monsoons and two of which are characterized as inter-monsoons^4^, so we did not include data from this study in our seasonal comparisons. However, we did include the fact that in Sri Lanka the majority of attacks occurred during the dry season. If a study reported the number of attacks per season, we used those numbers accordingly. If, however, the number of attacks was reported by month rather than season, we used the following dates to regroup them for Central India: summer (March–June), monsoon (July–October) and winter (November–February). Gujarat’s seasons are slightly different from those of central India, and therefore the following dates were used: summer (March–May), monsoon (June–September) and winter (October–February). Rajpurohit and Krausman^16^ documented bear attacks by month for both Madya Pradesh and the Bilaspur North Forest Division, a subregion within Madya Pradesh, so we treated these two areas separately.

The time-of-day of the attack was not always reported similarly between studies. We standardized this variable across studies by reclassifying the time-of-day into four categories: (1) dawn (twilight), (2) daylight, (3), dusk (twilight) and (4) dark. If studies used these categories, we used them as is. If, however, the time of day was given in two-hour increments, as reported by three different studies^17–19^, we regrouped the data as (1) dawn (twilight) 04:00–07:59, (2) daylight 08:00–15:59, (3) dusk 16:00–19:59 and dark 20:00–03:59., based on the website timeanddate.com (timeanddate.com), specifically the Sunrise and Sunset Calendar (time and date/sun). The time-of-day information from the Gujarat study^20^ was not used in this study because the data was not presented in a comparable way to the other studies.

### Analysis

To test for significant variation in the number of bear attacks by region according to season and time of day, we used the Chi-squared test, comparing expected and observed attack frequencies. We created a set of expected values by redistributing row totals across the 24-h day proportionally. Chi-squared test expected values represented our null hypothesis that time of day had no influence on the frequency of sloth bear attacks across the region.

## Results

Our analysis of regional variations in season and diel timing was based on 1,778 sloth bear attacks that were documented in eight studies. Seasonally we were able to assess 1,191 attacks (Table 2) and compare these findings to our null hypothesis (Table 3). Based on diel timing we were able to asses 995 attacks (Table 4) and compare these findings to our null hypothesis (Table 5).

**Table 2:** Sloth bear attack incidents by location and season.

Location	Summer	Monsoon	Winter	Total
Madya Pradesh	108	128	71	307
Bilaspur North	26	53	28	107
Chhattisgarh	37	74	26	137
Gujarat	16	11	36	63
Odisha	51	91	59	201
Kanha–Pench corridor	67	58	41	166
Maharashtra	18	26	7	51
Deccan Plateau	49	34	76	159
Total	372	475	344	1191

**Table 3:** A comparison of actual (observed) and expected seasonal attack counts by location using the Chi-square analysis.

Location	Summer	Monsoon	Winter
Madya Pradesh	↑	↑	↓
Bilaspur North	↓	↑	↓
Chhattisgarh	↓	↑	↓
Gujarat	↔	↓	↑
Odisha	↓	↑	↓
Kanha–Pench corridor	↑	↑	↓
Maharashtra	↑	↑	↓
Deccan Plateau	↓	↓	↑

A down arrow indicates lower than expected values; an up arrow indicates higher than expected and a horizontal (two headed) arrow means no difference.

**Table 4:** Sloth bear attacks by location and time of day.

Location	Morning	Day	Evening	Night	Total
Chhattisgarh	62	49	19	5	135
Sri Lanka	30	211	16	14	271
Odisha	94	41	42	15	192
Kanha–Pench corridor	29	104	24	9	166
Maharashtra	9	37	5	0	51
Deccan Plateau	23	40	35	82	180
Total	247	482	141	125	995

**Table 5:** A comparison of actual (observed) and expected time of day attack counts by location using the Chi-square analysis.

Location	Dawn	Day	Dusk	Night
Chhattisgarh	↑	↔	↔	↓
Sri Lanka	↔	↑	↓	↓
Odisha	↑	↔	↔	↓
Kanha–Pench corridor	↔	↑	↔	↓
Maharashtra	↔	↑	↔	↓
Deccan Plateau	↔	↔	↔	↔

A down arrow indicates lower than expected values; an up arrow indicates higher than expected and a horizontal (two headed) arrow means no difference.

### Season

**Figure 3:**
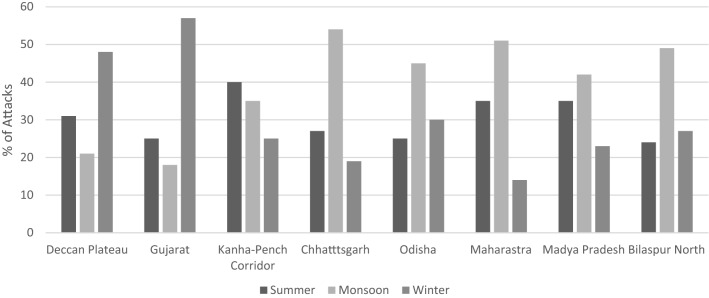
Seasonal percentages of attacks per region as reported in eight locations.

Compared to our null hypothesis, that there is no seasonality to sloth bear conflict on the Deccan Plateau, we found a higher-than-expected number of attacks in winter (48%), a lower-than-expected number of attacks during summer (31%), and significantly fewer attacks during monsoon season (21%: Fig. 3). In contrast, studies that occurred in central India had a higher-than-expected number of attacks during the monsoons and, with the exception of Odisha, a less than expected number of conflicts in the winter. Attack rates in summer varied greatly between study sites and a clear trend was not evident. However, the number of attacks in the Kanha-Pench Corridor was much higher than in the other study areas. Gujarat was the only area that, like the Deccan Plateau, showed an increase in the number of attacks during winter (57%). Gujarat, also like the Deccan Plateau, had the fewest number of attacks during the monsoon season (17%).

The Chi-squared test showed significant deviation within the table (*χ*^2^ = 5921, df = 5, *P* < 0.001) across rows and down columns for seasonal attack frequency across the 8 study areas. A graphical representation of individual count values by location and time of day, as compared to expected values, demonstrate the variation in attack patterns across India (↔ for no variation from expected values, (↑) for values higher than expected, and (↓) for values lower than expected).

### Time of day

**Figure 4:**
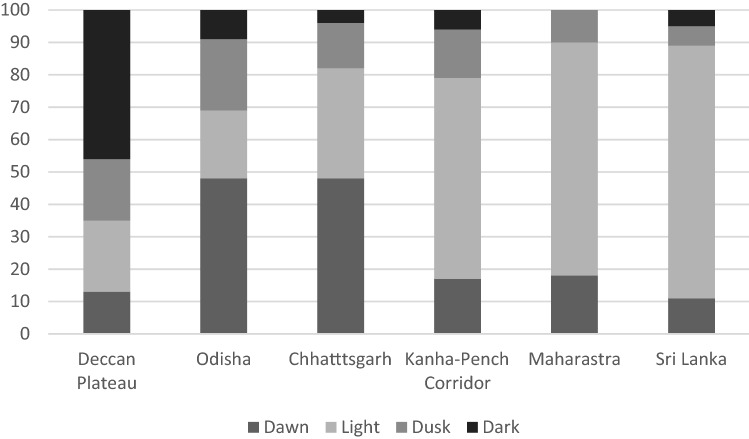
The percentages of sloth bear attacks by time of day and location.

Bear attacks on the Deccan Plateau were not affected by time of day, unlike any other regions we analyzed. The Deccan Plateau was also the only area where the number of attacks after dark was higher than at any other time (Fig. 4). Some areas, such as the Kanha-Pench corridor, Sri Lanka, and Maharashtra, reported more attacks during the day and fewer at night. Chhattisgarh shared similar deviations with respect to night being less than expected. Given these different patterns we ask why they varied so differently between regions.

The Chi-squared test revealed significant deviation within the table (*χ*^2^ = 666, df = 5, *P* < 0.001) across rows and down columns. A representation of individual count values by location and time of day, as compared to expected values, demonstrate the variation in attack patterns across India (↔ for no variation from expected values, (↑) for values higher than expected, and (↓) for values lower than expected). Upon analysis of the chi-square values for each cell, those highlighted contributed largely to significance.

## Discussion

### Seasonality of human–bear conflict

On the Deccan Plateau and Gujarat, most sloth bear attacks occurred in winter, which differs significantly from the seasonality of attacks reported by other studies. Unlike other study areas, people on the Deccan Plateau and in Gujarat are more active in the forest in winter when monsoons and crop harvests have ended. The higher incidence of attacks during monsoons in central India correlates with the increased presence of people farming and protecting crops from cattle depredation, as well as from bears and other wildlife species grazing in nearby forested areas^5, 16–18^. The Kanha–Pench Corridor study was the only one which documented an increase in sloth bear attacks during summer. This increase is concurrent with an increase of people in the forest that collect mahua flower (*Madhuca* spp) and tendu leaf (*Diospyros* spp)^19^. In Sri Lanka, most attacks occurred in the dry season, coincident with the highest levels of human activity in forested areas. People in Sri Lanka enter forests for alternative sources of income as agriculture activity declines during the dry season^4^.

Across all studies, the majority of sloth bear attacks are correlated with the time of year when human activity is greatest in bear habitat. However, the time of year that the peak of human activity occurs in sloth bear habitat varies by region. We conclude that the seasonal activity of bears plays a much smaller role on attack rates than the seasonal activity of humans. Consistent with findings in other studies, human incursion into bear habitat is the primary factor responsible for precipitating conflict^21^.

### Time of day influences on human–bear conflict

Most studies attributed the time of day that attacks occurred to when most humans were active in the forest^4, 17–20^. However, the Deccan plateau differed in that the majority of attacks occurred after dark when fewer people were active in or near the forest. Working in agricultural areas after dark is a more common practice on the Deccan Plateau than for the other study areas due to the availability of electricity and artificial lighting, though even with artificial lighting human activity after dark on the Deccan Plateau is still substantially less than during daytime. While a contributing factor, we do not feel that the increase in nighttime activity on the Deccan Plateau fully explains the significant increase in attacks during that time period as compared to other areas. We suspect that sloth bear activity patterns on the Deccan Plateau, and how bears use their environment, accounts for the shift in attack timing.

Sloth bears, though potentially active throughout the day, are predominately crepuscular and nocturnal^17, 22–24^. During daytime, sloth bears seek shelter in naturally occurring caves, crevices between big boulders, the spaces between tree roots, beneath fallen trees, or under bushes^1, 25–28^. On the Deccan Plateau, however, sloth bears utilize rocky caves almost exclusively for daytime denning^29^. A cave reduces chance encounters with people and predators while providing a modicum of security, hence the lower incident rate for areas with naturally occurring caves.

Conversely, studies conducted in Sri Lanka, Maharashtra and the Kanha-Pench corridor documented more attacks during daytime when people are more active but sloth bears are less active^4, 5, 19^. Large areas where sloth bears are located in Sri Lanka do not have caves for resting, though they do have dense vegetation and tree cavities (S. Ratnayeke, personal communication July 28, 2020). The Dnyanganga Wildlife Sanctuary, in the state of Maharastra, is mostly lower plains forest without rocky caves (N. Dharaiya, personal communication June 25, 2020). The Kanha-Pench corridor landscape is largely comprised of sal (*Shorea* spp) and teak (*Tectona* spp) forests largely devoid of caves^30^. The role of caves in minimizing daylight sloth bear attacks may be best exemplified by an attack in Sri Lanka as quoted in Ratnayeke et al.^4^:

“I was following two of my companions and saw a black form lying at the foot of a clump bushes, about 10 m from me. I called out to my companions. Before I knew it, the impact of the charging bear knocked me off my feet. It happened so fast, I didn’t see the bear coming… just dust, flying leaves, and the screams and roars of the bear.”

Had this bear been in a cave rather than the shade of a bush, it likely would not have felt threatened and reacted defensively. We speculate that during daylight on the Deccan Plateau, sloth bears rest securely within a cave and are not threatened by humans passing nearby. We know that farmers and livestock herders work in relatively close proximity to known den locations without fear of being attacked (S. Shanmugavelu, pers. observation). Clearly, caves afford a level of protection and separation that benefits both bears and humans. Consequently, we suggest this is the most likely explanation as to why there are relatively few attacks on the Deccan Plateau during daytime.

### Season and sloth bear safety messaging

Bear attack research and safety messaging often recognizes a seasonal component^17–20, 31^ (e.g., more sloth bear attacks occur during the monsoon season than during other seasons). Sloth bears are active year-round, and the rate of attacks is strongly correlated with the level of human activity in the forest. Similarly, in Alaska, Smith and Herrero^32^ reported that human-brown bear conflicts were strongly seasonal in their occurrence. Additionally, they reported that attacks occurred most often when both people and bears vied for the same resource, such as salmon or ungulates. Farther north, human-polar bear conflict peaks when bears are on land awaiting freeze up in the fall^33^. Not infrequently, sloth bear safety messaging amounts to little more than general statements such as “when in the forest or in sloth bear country be aware”. In other words, an individual’s odds of being attacked by a sloth bear while in the woods may not significantly vary regardless of season. But, where it has been found to vary by season, this information should be conveyed to the public.

### Time of day and sloth bear safety messaging

Sloth bear research and safety messaging often reports and warns of the “most dangerous” time or times of the day to be active in the forest^17–20, 31, 34^. Sloth bear attacks, like grizzly bear or American black bear attacks^33^, can occur anytime, day or night^6^. However, due to an abundance of naturally occurring caves on the Deccan Plateau, stumbling across a sleeping sloth bear mid-day is much less likely to occur than it is in Sri Lanka or in the Kanha-Pench corridor. Therefore, regional sloth bear safety messaging should acknowledge this significant difference which will promote bear safety.

The Corbett Foundation^31^ and Dharaiya et al.^34^ do an admirable job of focusing their safety messaging to a specific regional group of people in their respective publications. This type of regional messaging is necessary for optimizing sloth bear safety messaging efficacy. However, there is also value to non-site-specific sloth bear safety messaging. The short film “Living with Sloth Bears”^35^ intentionally addresses general safety messaging that applies to sloth bears across their entire range. Consequently, in the making of this film, we purposely avoided referring to the timing of attacks, seasons or time of day, or other aspects of human-bear conflict because we were aware of significant differences with respect to these variables between locations.

Yet another aspect of bear safety messaging is to keep it simple so that a person, under duress, will remember what to do in the event of a bear encounter Attempting to recall the details of an extended message, especially when being threatened by a bear, can be difficult, if not impossible. Therefore, the trend has been to keep bear messaging as simple as possible and we agree with it. However, teaching people that work in bear habitat the most likely times of day encounters occur can be beneficial. In summary, there is a time and place to provide detailed information that is regionally specific, and other situations in which to keep messaging simple.

### Sloth bear denning ecology on the Deccan Plateau and its role in human–bear conflict

The Deccan Plateau is known as high quality sloth bear habitat, as evidenced by the relatively high density of bears in this area (S. Shanmugavelu, pers. observation). While there is ample food on the Deccan Plateau, the abundance of caves there sets it apart from other areas within the specie’s range. Sloth bears use only caves or cave-like structures on the Deccan Plateau for resting (Shanmugavelu et al. In Print). Caves provide protection from the elements, such as the heat of the day or severe storms, as well as protection from potential predators. Sloth bears do not have many predators and while a cub or very young bear may be at risk from leopards (*Panthera pardus*) or wolves (*Canis lupes pallipes*), the only natural predator of adult sloth bears is the Bengal tiger (*Panthera tigris tigris*). Tiger scat studies revealed that sloth bears can comprise up to 2% of a their diet^36–39^. Tigers no longer occur on the Deccan Plateau, but the abundance of caves in the area undoubtedly historically benefited sloth bears, perhaps facilitating a higher density than would have been otherwise attainable. Presently, however, an increase in human population and habitat loss represents greater threat to the species.

## Conclusions

Bear safety messaging often differs regionally. Periodically, these differences have been the source of discussion and disagreement between bear biologists, park managers and the public. Should bear safety messaging be more standardized so that there is less confusion, or are regional differences in human-bear conflict important to incorporate? While sloth bear safety messaging is still in its early stages of development compared to that of American black and brown bears, results of this study suggest that incorporation of regional differences is important to optimize human safety. While some regional differences in human-bear conflict are due to varying modes of human activity, in other instances differences in messaging are reflective of variation in bear activity and how the species has evolved to modify behaviors to best fit environmental variation. In seeking to ensure human safety in bear habitat, we see variation in messaging as key to helping better prepare people to avoid bear conflict.
